# Timing of Endoscopic Biliary Drainage for Acute Cholangitis Associated with Common Bile Duct Stones in Elderly Patients: A Single-Center Retrospective Cohort Study

**DOI:** 10.3390/jcm15135153

**Published:** 2026-07-02

**Authors:** Hironao Ichikawa, Akinori Maruta, Kaori Koide, Hiroki Taniguchi, Susumu Imai, Tomomichi Matsushita, Junko Shiroko, Takuji Iwashita, Masahito Shimizu

**Affiliations:** 1 Department of Internal Medicine, Japanese Red Cross Takayama Hospital, Takayama 506-8550, Gifu, Japan; ichi.hiro.m.0814@gmail.com (H.I.);; 2 First Department of Internal Medicine, Gifu University Hospital, Gifu 501-1194, Gifu, Japan; 3 Department of Gastroenterology, Shiga University of Medical Science Hospital, Otsu 520-2192, Shiga, Japan

**Keywords:** acute cholangitis, early biliary drainage, late biliary drainage, elderly patients, ERCP

## Abstract

**Background/Objectives:** The optimal timing for biliary drainage in elderly patients remains controversial. This study compared the clinical outcomes of early versus late biliary drainage in elderly patients diagnosed with acute cholangitis associated with common bile duct stones (CBDS). **Methods:** This single-center, retrospective cohort study compared early (within 24 h of admission) versus late (>24 h after admission) biliary drainage using endoscopic retrograde cholangiopancreatography (ERCP) in 143 elderly patients diagnosed with acute cholangitis, who were divided into early and late groups, respectively. **Results:** There were no statistical differences in patient characteristics between the early and late drainage groups. Although the time to clinical success was significantly shorter in the early drainage group, there were no significant differences in persistent organ failure, length of hospital stay, in-hospital and 30-day mortality, and ERCP-related adverse events (AEs) between the early and late groups. In the late group, the clinically worsened group tended to exhibit higher white blood cell counts and lower platelet counts on admission. **Conclusions:** Time to clinical success was significantly shorter in the early drainage group than in the late group. No statistically significant differences in persistent organ failure, length of hospital stay, in-hospital and 30-day mortality, and ERCP-related adverse AEs were detected between early and late biliary drainage in elderly patients with acute cholangitis associated with CBDS.

## 1. Introduction

Cholangitis is caused by bacterial infection of the bile duct and is often triggered by obstruction or increased intraductal pressure. Severe acute cholangitis can be complicated by septic shock and disseminated intravascular coagulation, potentially resulting in a fatal course. According to previous reports [1,2], cholangitis has a mortality rate of 3.5–14.2% when it becomes severe, thus making appropriate diagnosis and prompt treatment crucial.

According to the Tokyo Guidelines 2018 (TG18) [3] for the treatment of acute cholangitis, for mild cases, if antibiotic therapy is ineffective, biliary drainage should be considered; for moderate cases, biliary drainage should be started with prompt consideration; and for severe cases, emergency biliary drainage should be considered while providing organ support. These recommendations indicate that early biliary drainage becomes increasingly important as the severity of acute cholangitis increases. However, despite these recommendations, the optimal timing of biliary drainage remains controversial.

In a retrospective comparative study using a database of patients with acute cholangitis of all severities, Mulki et al. [4] divided 4507 patients into early (endoscopic retrograde cholangiopancreatography (ERCP) ≤ 48 h) and delayed (ERCP > 48 h) drainage groups. The investigators reported significantly lower 30-day mortality in the early group compared with the delayed group (1.5% vs. 3.3%, respectively; adjusted odds ratio [aOR] 0.48 (95% confidence interval (CI) 0.33–0.69; *p* < 0.001), demonstrating the efficacy of early biliary drainage for acute cholangitis. Conversely, Aboelsoud et al. [5] examined the impact of biliary drainage timing in 177 patients who underwent biliary drainage for acute cholangitis of all severities. The authors found no significant difference in in-hospital mortality between the early (within 24 h) and delayed (>24 h) drainage groups. While some reports indicate that early biliary drainage reduces in-hospital mortality [6,7,8], others have reported no significant difference [5,9], making the timing of biliary drainage for acute cholangitis controversial. Several previous studies [1,4,6] have used 12, 24, and 48 h thresholds to define early biliary drainage. In the present study, we selected 24 h as the cutoff because it is one of the most commonly used definitions in previous studies [6,9] and clinical practice.

With the rapid aging of the global population, the prevalence of acute cholangitis among elderly patients has been increasing steadily. Elderly patients more commonly present with multiple comorbidities, reduced physiological reserve, frailty, and impaired immune function, all of which may contribute to adverse clinical outcomes and heightened susceptibility to organ failure and sepsis. Furthermore, the high prevalence of cardiovascular and pulmonary comorbidities may increase the risks associated with emergent endoscopic intervention and procedural sedation. Age ≥75 years is included in the diagnostic criteria for moderate cholangitis in TG18, and advanced cholangitis is considered to be a risk factor for disease progression. Regarding endoscopic treatment for acute cholangitis associated with common bile duct stones (CBDS), emergent biliary drainage cannot always be performed during after-hours periods, such as nighttime. Various reports have addressed the timing of biliary drainage for acute cholangitis. Evidence specifically targeting elderly patients remains limited, as most prior studies enrolled heterogeneous age-group populations, leaving the clinical impact of emergent biliary drainage in this demographic incompletely characterized. In clinical practice, physicians frequently exercise caution when considering emergent biliary drainage in elderly patients, given the heightened risks associated with sedation-related adverse events (AEs), cardiopulmonary complications, anticoagulant therapy management, cognitive impairment, and frailty. Accordingly, the risk–benefit balance between the therapeutic advantages of prompt source control and the procedural risks inherent to emergent biliary drainage may differ substantially between elderly and younger patient populations.

Consequently, whether the benefits of early biliary drainage observed in the general population can be directly extrapolated to elderly patients remains unclear. Therefore, the present study investigated the impact of endoscopic biliary drainage timing on treatment outcomes in elderly patients with mild-to-moderate acute cholangitis associated with CBDS.

## 2. Patients and Methods

### 2.1. Study Design and Patient Selection

This retrospective cohort study was conducted at a single center. A database housing information for all ERCP procedures performed at the Japanese Red Cross Takayama Hospital (Takayama, Gifu, Japan) between April 2015 and July 2023 was used to identify patients >75 years of age who underwent ERCP for mild or moderate acute cholangitis associated with CBDS. The exclusion criteria were as follows: history of endoscopic therapy for CBDS; presence of malignant or benign common bile duct strictures; and history of upper gastrointestinal or biliary tract surgery, except for gastrectomy with Billroth I reconstruction. This study was conducted in accordance with the principles of the Declaration of Helsinki, and the study protocol was approved by the Institutional Review Board of the authors’ center (R05-04).

### 2.2. ERCP Procedure

ERCP was performed using a standard duodenoscope (TJF-290V or 260V, Olympus, Tokyo, Japan). After successful cannulation of the bile duct, cholangiography was performed to determine the number and size of CBDS. Endoscopic sphincterotomy (EST), endoscopic papillary balloon dilation (EPBD), or endoscopic papillary large balloon dilation (EPLBD) was performed depending on the size and number of CBDS and patient condition. CBDSs were removed using a balloon catheter (Extractor ProRX, Boston Scientific, Marlborough, MA, USA) and/or a basket catheter (Memory Basket Eight Wire, Cook Medical Japan G.K., Tokyo, Japan). If the CBDSs were too large for extraction, mechanical lithotripsy (LithoCrush V, Olympus, Tokyo, Japan) was performed for lithotomy. In cases in which complete CBDS removal was difficult in a single session, a plastic stent was placed, and another ERCP was scheduled to manage the CBDS. In patients undergoing antiplatelet or anticoagulant therapy, or those with severe inflammation, a plastic stent was placed without removing the CBDS, and another ERCP was scheduled to manage the CBDS. The timing of biliary drainage was determined by the attending physician based on the patient’s clinical condition, endoscopic availability, and hospital resources. Laboratory tests performed 2–4 days after ERCP were evaluated. Patients were followed until hospital discharge. Clinical outcomes, including mortality, length of hospital stay, and ERCP-related AEs, were evaluated during hospitalization.

### 2.3. Outcomes, Definitions and Statistical Analysis

The primary endpoint was the time to clinical success, with biliary drainage performed within 24 h after admission (early group) and >24 h later (late group). The secondary endpoints were persistent organ failure, length of hospital stay, in-hospital mortality, 30-day mortality, and ERCP-related AEs. Technical success was defined as successful endoscopic biliary drainage or endoscopic removal of the CBDS. The diagnosis and severity of acute cholangitis were evaluated according to the TG18 guidelines [3]. ERCP-related AEs were defined based on the lexicon for endoscopic AEs of the American Society of Gastroenterological Endoscopy [10]. Clinical success was defined as improvement in symptoms (abdominal pain and fever) and improvement in serum liver enzyme levels by ≥50%. Persistent organ failure after ERCP was defined as hypotension requiring catecholamines, respiratory failure requiring mechanical ventilation, or acute kidney injury (1.5-fold increase in serum creatinine level from baseline or need for dialysis) lasting >48 h. Clinical worsening was defined as progression of TG18 severity classification during the waiting period before ERCP. During the waiting period, blood tests were repeatedly performed every 1–3 days, and the severity was reassessed as appropriate based on clinical and laboratory findings.

Fisher’s exact test was used to compare proportions of categorical variables, and the Mann–Whitney U test was used to compare continuous variables between the early and late groups. Continuous variables are expressed as median (interquartile range [IQR]). Clinical success between the two groups was estimated using the Kaplan–Meier method and compared using the log-rank test. All statistical analyses were performed using EZR (Saitama Medical Center, Jichi Medical University, Saitama, Japan), a graphical user interface for R version 4.5.2 (R Core Team; Foundation for Statistical Computing, Vienna, Austria).

## 3. Results

### 3.1. Patient Characteristics

Patient characteristics are summarized in Table 1.

A total of 143 patients were enrolled, with 102 and 41 patients in the early and late groups, respectively. Patient data collected at admission included age, sex, Charlson comorbidity index (CCI), white blood cell (WBC) count, platelet count, albumin level, aspartate aminotransferase (AST) level, total bilirubin level, C-reactive protein (CRP), antiplatelet/anticoagulant therapy, primary disease, severity of cholangitis, and gallbladder stones. There were no significant differences between the early and late groups; however, WBC count was significantly higher in the early group than that in the late group (*p* = 0.044).

### 3.2. Clinical Outcomes

ERCP-related outcomes are reported in Table 2.

The median time for biliary drainage after admission was 4 h (IQR 2–14.8 h) in the early group and 46 h (IQR 28–69 h) in the late group (*p* < 0.01). The rate of initial biliary drainage without removing CBDS was 52.9% (54/102) in the early group and 19.5% (8/41) in the late group (*p* < 0.01). The median size of CBDS was 8.6 mm (IQR 5–12 mm) in the early group and 8 mm (IQR 5–11 mm) in the late group (*p* = 0.313). The median bile duct diameter was 10 mm (IQR 8–14 mm) in the early group and 11 mm (IQR 8.9–13 mm) in the late group (*p* = 0.98). The median procedure time was 20 min (IQR 13.3–34.8 min) in the early group and 20 min (IQR 15–39 min) in the late group (*p* = 0.542). The median number of interventions was 1 (IQR 1–2) in the early group and 1 (IQR 1–2) in the late group (*p* = 0.041). The technical success rate was 93.1% (95/102) in the early group and 90.2% (37/41) in the late group (*p* = 0.512). ERCP-related AEs occurred in 3.9% (4/102) of patients in the early group and in 7.3% (3/41) of patients in the late group (*p* = 0.409).

Prognostic outcomes are summarized in Table 3.

**Figure 1:**
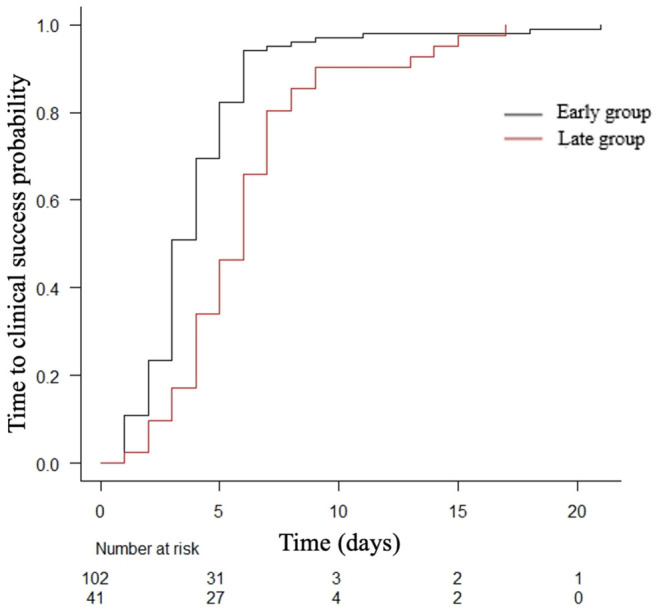
Kaplan–Meier curves of time to clinical success of the early group and the late group. The median time to clinical success estimated by Kaplan–Meier method was 3 days in the early group and 6 days in the late group (*p* < 0.01, log-rank test).

The clinical success rates in the early and late groups were 100% (102/102) and 100% (41/41), respectively (*p* = 1). The median time to clinical success in the early and late groups was 3 days (IQR 3–5 days) and 6 days (IQR 4–7 days), respectively (*p* < 0.01), and significantly shorter in the early group than in the late group. Figure 1 shows the cumulative probabilities of clinical success estimated using the Kaplan–Meier method, and the median time to clinical success was significantly shorter in the early group than in the late group (HR 0.54, 95% confidence interval 0.37 to 0.79; log-rank *p* < 0.01).

Persistent organ failure occurred in 8.8% (9/102) of patients in the early group and 2.4% (1/41) of patients in the late group (*p* = 0.282). The median WBC count 2–4 days after ERCP was 5900/μL (IQR 4505–7925/μL) in the early group and 5100/μL (IQR 4370–6700/μL) in the late group (*p* = 0.199). The median CRP level 2–4 days after ERCP was 4.51 mg/dL (IQR 3.04–9.38 mg/dL) in the early group and 2.46 mg/dL (IQR 0.91–8.33 mg/dL) in the late group (*p* < 0.01). However, the median WBC count and CRP 1 week after ERCP were not significantly different between the early and late groups. The median length of hospital stay was 11 days (IQR 8–17 days) in the early group and 11 days (IQR 7–15 days) in the late group (*p* = 0.927). The in-hospital mortality rate was 3.9% (4/102) in the early group and 2.4% (1/41) in the late group (*p* = 1). The 30-day mortality rate was 2.9% (3/102) in the early group and 2.4% (1/41) in the late group (*p* = 1).

Baseline characteristics of patients in the late group are summarized in Table 4.

Patients who experienced clinical worsening of cholangitis (clinically worsened group [n = 7]) were compared with those who experienced improved clinical severity of cholangitis (clinically improved [n = 34]) while awaiting ERCP based on the TG18 guidelines. Age, sex, CCI, albumin, AST, total bilirubin levels, serum creatinine (Cr), antiplatelet/anticoagulant therapy, primary disease, cholangitis severity, and gallbladder stones were not significantly different between the two groups. However, the median WBC count was 9300/μL (IQR 8550–14,125/μL) in the clinically worsened group and 7705/μL (IQR 5253–21,000/μL) in the clinically improved group (*p* = 0.127). The median platelet count was 16 × 10^3^/μL (IQR 14–18.2 × 10^3^/μL) in the clinically worsened group and 19.4 × 10^3^/μL (IQR 15.5–23.7 × 10^3^/μL) in the clinically improved group (*p* = 0.166). In the late group, the clinically worsened group tended to exhibit higher WBC counts and lower platelet counts on admission.

## 4. Discussion

The results of this single-center retrospective study revealed that the time to clinical success was significantly shorter in the early biliary drainage group than in the late group. However, no significant differences were observed between the two groups in terms of technical or clinical success, prognosis (presence or absence of organ dysfunction, length of hospital stay, 30-day mortality rate, and in-hospital mortality rate), or ERCP-related AEs.

Elderly patients have a higher prevalence of comorbidities and exhibit a decline in physical and mental functions, resulting in distinct characteristics of acute cholangitis that differ from those observed in younger patients. First, elderly patients with acute cholangitis are more likely to have underlying diseases than their non-elderly counterparts. In a retrospective comparative study involving 207 patients who underwent ERCP for acute cholangitis, Tohda et al. [11] divided patients into elderly (≥80 years) and non-elderly (<80 years) groups. Elderly patients with acute cholangitis exhibited a significantly higher overall prevalence of primary diseases compared with the non-elderly (91.2% vs. 67.6%, respectively; *p* < 0.05). Furthermore, detailed analysis revealed that the rates of hypertension, ischemic heart disease, cerebrovascular disease, dementia, chronic obstructive pulmonary disease, and malignancy were significantly higher in the elderly group. Second, elderly patients may not present with the typical symptoms of acute cholangitis compared with their non-elderly counterparts. Additionally, due to declines in physical function, hospital visits are often delayed, leading to the discovery of conditions in a more severe state. Lee et al. [12] conducted a retrospective comparative study of 260 patients with acute cholangitis, dividing them into two groups: ≥75 years and <75 years of age. In the elderly group (≥75 years), the distribution of disease severity was mild (18.7%), moderate (54.5%), and severe (26.9%), whereas in the non-elderly group (<75 years), it was mild (59.5%), moderate (31%), and severe (9.5%). Furthermore, the elderly group exhibited significantly higher disease severity (*p* < 0.01). These findings suggest that elderly patients with acute cholangitis may present to hospital later and are more likely to be diagnosed at a more severe stage of the disease. Third, hospitalization of elderly patients may lead to the worsening of dementia and a decline in the activities of daily living. Sugiyama et al. [13] conducted a retrospective comparative study of treatment outcomes in patients with acute cholangitis associated with CBDS who underwent emergency biliary drainage, dividing them into elderly (≥80 years [n = 37]) and non-elderly (<80 years [n = 154]) groups. They reported neurological abnormalities in 24.3% of the elderly group and 6.5% of the non- elderly group, with a significantly higher incidence in the elderly group (*p* < 0.01). Appropriate biliary drainage aimed at early discharge may potentially improve post-discharge quality of life.

Park et al. [14] conducted a retrospective study comparing the clinical characteristics of 331 patients with acute cholangitis who underwent biliary drainage and divided them into elderly (75–80 years [n = 156]) and very elderly (≥81 years [n = 176]) groups. The study identified disease severity (severe > moderate/mild) and the success or failure of drainage as factors associated with 30-day mortality in elderly and very elderly patients with acute cholangitis and the overall mortality rate was 1.5%. The hospital stay was shorter in the urgent group than in the elective group. In the present study, the early drainage group exhibited a significantly shorter time to clinical success than the late group, supporting the efficacy of early drainage. Farooq U et al. [15] evaluated the efficacy and safety of TG18 in patients with acute cholangitis across all age groups. Of the 137,100 patients, 93,365 (68.09%) had non-severe cholangitis, and 43,735 (31.91%) had severe cholangitis. The patients were classified into three categories: 18–64 years, 65–79 years, and 80 years and older. For severe acute cholangitis, the in-hospital mortality was 6.06% in patients who underwent emergent ERCP compared to 13.67% in those who underwent ERCP after 24 h. Similarly, urgent ERCP resulted in a decreased in-hospital mortality from 2.33% to 0.77% in patients with mild or moderate acute cholangitis. Emergent ERCP resulted in decreased mortality in all age groups for both severe and non-severe acute cholangitis across all age groups compared to when performed after 24 h.

Conversely, Hakuta et al. [16] conducted a retrospective comparative study of clinical outcomes in 299 patients with mild to moderate acute cholangitis, dividing them into early (n = 201; median age, 73 years [IQR 65–83 years]) and delayed (n = 98; median age, 74 years [IQR 66–80 years]) drainage groups. The early group underwent biliary drainage within 12 h, whereas the delayed group underwent biliary drainage >12 h. The investigators reported in-hospital mortality rates of 0.5% (1/201) and 0% (0/98) in the early and delayed drainage groups, respectively, with no statistical difference between the two groups. In the present study, which was also limited to mild-to-moderate acute cholangitis, no differences were observed between the two groups in terms of organ dysfunction, hospital stay, and 30-day and in-hospital mortality. These results suggest that, in elderly patients with mild-to-moderate acute cholangitis, the prognosis did not differ significantly between the early and late drainage groups.

The effect of late drainage on clinical course and prognosis remains largely unclear, with concerns raised regarding the worsening of acute cholangitis during the wait period for biliary drainage. In the present study, the rate of cholangitis severity worsening during the wait period for biliary drainage was 17.1% (7/41). When patients were divided into a clinically worsened group (n = 7) and a clinically improved group (n = 34), the clinically worsened group tended to exhibit higher WBC counts and lower platelet counts at admission. Based on these findings, higher WBC counts and lower platelet counts at admission may serve as potential risk factors for worsening of acute cholangitis in elderly patients. However, because of the limited number of clinically worsened cases and the retrospective nature of this study, these findings should be interpreted cautiously.

Based on the results of the present study, early biliary drainage for acute cholangitis associated with CBDS in elderly patients appears to be beneficial because it may shorten the time to clinical success. No statistically significant differences in mortality and length of hospital stay were detected between the early and late drainage groups. However, these findings should be interpreted cautiously because of the retrospective design, limited sample size, and potential confounding factors. Nevertheless, in patients presenting with an elevated WBC count or low platelet count at admission, there is a possibility of worsening cholangitis severity during the wait period, thus necessitating the consideration of early biliary drainage.

The present study had several limitations, the first of which was its retrospective single-center design, which may have introduced selection bias, and lacked a standardized treatment strategy. Second, this was a small-scale study with a limited number of cases. Because of the limited number of the patients in the late group, the absence of statistically significant differences in mortality, persistent organ failure, and ERCP-related AEs between the two groups should be interpreted cautiously. Third, the study only included patients who underwent endoscopic biliary drainage for acute cholangitis; therefore, patients with acute cholangitis who received conservative treatment without biliary drainage and those who underwent other biliary drainage methods (e.g., percutaneous transhepatic biliary drainage and endoscopic ultrasound-guided biliary drainage) could not be evaluated. In addition, because this was a retrospective non-randomized study, substantial confounding by indication may have been present. The timing of biliary drainage was determined by the attending physicians based on the patients’ clinical condition, disease severity, procedural availability, and overall stability. Therefore, unmeasured confounding factors may have influenced the observed outcomes despite similar TG18 severity classifications between groups. Finally, metabolic dysfunction associated steatotic liver disease (MASLD) status was not systematically assessed in the present study. Therefore, the potential influence of underlying MASLD on the clinical course and outcomes of acute cholangitis could not be evaluated. Future large-scale prospective multicenter studies are needed to establish evidence-based recommendations regarding the optimal timing of biliary drainage in elderly patients with acute cholangitis.

In conclusion, although time to clinical success was significantly shorter in the early drainage group than that in the late group and no statistically significant differences in persistent organ failure, length of hospital stay, in-hospital and 30-day mortality, and ERCP-related AEs were detected between early and late biliary drainage in elderly patients with acute cholangitis associated with CBDS, these findings should be interpreted cautiously because of the retrospective design and limited sample size. Further large-scale prospective multicenter studies are needed to determine the optimal timing of biliary drainage in this population.

## Figures and Tables

**Table 1:** Baseline characteristics of patients.

		Early Group *n* = 102	Late Group *n* = 41	*p*-Value
Age, yo	median (IQR)	85 (81–91)	86 (83–90)	0.83
Gender, men	n (%)	45 (44.1)	19 (46.3)	0.85
CCI	median (IQR)	6 (5–7)	6 (5–7)	0.70
WBC, /μL	median (IQR)	10,400 (7960–13,268)	8600 (6280–11,700)	0.04
Platelet, ×10^3^/μL	median (IQR)	18.0 (15.1–22.6)	17.3 (15.4–22.9)	0.81
Albumin, mg/dL	median (IQR)	3.4 (3.0–3.7)	3.5 (3.0–3.8)	0.85
AST, mg/dL	median (IQR)	262.5 (120.5–625.3)	223 (165.0–392.0)	0.55
Total bilirubin level, mg/dL	median (IQR)	2.25 (1.5–3.6)	2.5 (1.6–4.5)	0.79
CRP, mg/dL	median (IQR)	5.6 (1.4–8.5)	4.5 (1.4–7.6)	0.72
Antiplatelet/anticoagulant therapy	n (%)	40 (39.2)	10 (24.4)	0.12
Primary disease	n (%)	53 (52.0)	17 (41.5)	0.27
Severity				0.44
Mild Moderate	n (%)	68 (66.7) 34 (33.3)	24 (58.5) 17 (41.5)	
Gallbladder stone	n (%)	71 (69.6)	26 (63.4)	0.55

CCI, Charlson comorbidity index; CRP, C-reactive protein; IQR interquartile range; WBC, white blood cell.

**Table 2:** ERCP-related outcomes.

		Early Group *n* = 102	Late Group *n* = 41	*p*-Value
Time to biliary drainage, h	median (IQR)	4 (2–14.8)	46 (28–69)	<0.01
Initial drainage without removing CBDS	n (%)	54 (52.9)	8 (19.5)	<0.01
Maximum size of CBDS, mm	median (IQR)	8.6 (5–12)	8 (5–11)	0.31
Bile duct diameter, mm	median (IQR)	10 (8–14)	11 (8.9–13)	0.98
Procedure time, min	median (IQR)	20 (13.3–34.8)	20 (15–39)	0.54
Number of interventions	median (IQR)	1 (1–2)	1 (1–2)	0.04
Technical success	n (%)	95 (93.1)	37 (90.2)	0.51
ERCP-AEs	n (%)	4 (3.9)	3 (7.3)	0.41
PEP		3 (2.9)	3 (7.3)	
Others		1 (1)	0 (0)	

AEs, adverse events; CBDS, common bile duct stones; ERCP, endoscopic retrograde cholangiopancreatography; IQR, interquartile range; PEP, post endoscopic retrograde cholangiopancreatography pancreatitis.

**Table 3:** Prognostic outcomes.

		Early Group *n* = 102	Late Group *n* = 41	*p*-Value
Clinical success	n (%)	102 (100)	41 (100)	1.00
Time to clinical success, days	median (95% CI)	3 (3–4)	6 (4–6)	<0.01
Persistent organ failure (>48 h)	n (%)	9 (8.8)	1 (2.4)	0.28
WBC 2–4 days after ERCP, /μL	median (IQR)	5900 (4505–7925)	5100 (4370–6700)	0.12
CRP 2–4 days after ERCP, mg/dL	median (IQR)	4.51 (3.04–9.38)	2.46 (0.91–8.38)	<0.01
WBC one week after ERCP, /μL	median (IQR)	5900 (4900–7000)	6070 (4925–7125)	0.85
CRP one week after ERCP, mg/dL	median (IQR)	1.81 (0.93–3.45)	1.70 (0.72–3.51)	0.57
Length of stay, days	median (IQR)	11 (8–17)	11 (7–15)	0.93
In-hospital mortality	n (%)	4 (3.9)	1 (2.4)	1.00
30-day mortality	n (%)	3 (2.9)	1 (2.4)	1.00

95% CI; 95% confidential interval; CRP, C-reactive protein; IQR, interquartile range; WBC, white blood cell.

**Table 4:** Baseline characteristics of patients in the late group.

		Clinically Worsened Group *n* = 7	Clinically Improved Group *n* = 34	*p*-Value
Age, yo	median (IQR)	86 (85–86.5)	86 (82.3–91)	0.55
Gender, men	n (%)	4 (57.1)	15 (44.1)	0.69
CCI	median (IQR)	6 (5–7)	6 (5–7)	0.86
WBC, /μL	median (IQR)	9300 (8550–14,125)	7705 (5253–21,000)	0.13
Platelet, ×10^3^/μL	median (IQR)	16 (14–18.2)	19.4 (15.5–23.7)	0.17
Albumin, mg/dL	median (IQR)	3.5 (3.1–3.9)	3.5 (3.0–3.7)	0.81
AST, mg/dL	median (IQR)	349 (207–487)	220 (146–390)	0.42
Total bilirubin level, mg/dL	median (IQR)	3.0 (2.7–4.1)	2.2 (1.1–4.9)	0.36
Cr, mg/dL	median (IQR)	0.79 (0.71–1.00)	0.87 (0.74–1.13)	0.72
Antiplatelet/anticoagulant therapy	n (%)	3 (42.9)	11 (32.4)	0.33
Primary disease	n (%)	3 (42.9)	14 (41.2)	1.00
Severity				0.68
Mild Moderate	n (%)	5 (71.4) 2 (28.6)	19 (55.9) 15 (44.1)	
Gallbladder stone	n (%)	3 (42.9)	23 (67.6)	0.39

CCI, Charlson comorbidity index; Cr, creatinine; IQR interquartile range; WBC, white blood cell.

## Data Availability

The data presented in this study are available from the corresponding author upon reasonable request.

## Abbreviations

AEs, adverse events; CBDS, common bile duct stones; CCI, Charlson comorbidity index; EPBD, endoscopic papillary balloon dilation; EPLBD, endoscopic papillary large balloon dilation; ERCP, endoscopic retrograde cholangiopancreatography; EST, endoscopic sphincterotomy.
